# The Association between Endogenous Hair Steroid Hormones and Social Environmental Factors in a Group of Conscripts during Basic Military Training

**DOI:** 10.3390/ijerph182212239

**Published:** 2021-11-22

**Authors:** Asta Mažeikienė, Svajone Bekesiene, Dovilė Karčiauskaitė, Eglė Mazgelytė, Gerry Larsson, Tomas Petrėnas, Andrius Kaminskas, Jurgita Songailienė, Algirdas Utkus, Ramutė Vaičaitienė, Rasa Smaliukienė

**Affiliations:** 1 Department of Physiology, Biochemistry, Microbiology and Laboratory Medicine, Institute of Biomedical Sciences, Faculty of Medicine, Vilnius University, LT-03101 Vilnius, Lithuania; dovile.karciauskaite@mf.vu.lt (D.K.); egle.mazgelyte@mf.vu.lt (E.M.); gerry.larsson@fhs.se (G.L.); 2 General Jonas Zemaitis Military Academy of Lithuania, LT-10322 Vilnius, Lithuania; ramute.vaicaitiene@lka.lt (R.V.); rasa.smaliukiene@lka.lt (R.S.); 3 Department of Security, Strategy and Leadership (ISSL), Swedish Defence University, 65180 Karlstad, Sweden; 4 Department of Public Health, Inland Norway University of Applied Sciences, 2418 Elverum, Norway; 5 Department of Human and Medical Genetics, Institute of Biomedical Sciences, Faculty of Medicine, Vilnius University, LT-03101 Vilnius, Lithuania; tomas.petrenas@santa.lt (T.P.); andrius.kaminskas@santa.lt (A.K.); jurgita.songailiene@mf.vu.lt (J.S.); algirdas.utkus@mf.vu.lt (A.U.)

**Keywords:** hair steroid hormones, cortisol, cortisone, dehydroepiandrosterone, testosterone, military conscripts, military training social environmental factors, chronic stress biomarkers

## Abstract

This study aimed to analyse the association between endogenous hair steroid hormones as reliable biological indicators of an individual’s stress level and the social environmental factors experienced during military training that are manifested at the beginning of compulsory military service. Hair steroid hormone concentrations—cortisol, cortisone, dehydroepiandrosterone (DHEA), and testosterone—in a group of 185 conscripts were measured using the ultra-high performance liquid chromatography-tandem mass spectrometry method. Six subjective social environmental factors in the military—attitude towards the military and military service, adaptation to the military environment, team, task, and norm cohesion, as well as psychological (un)safety in the group—were evaluated using military-specific research questionnaires. Weak but significant negative correlations were identified between cortisol and adaptation (r = −0.176, *p* < 0.05), attitude (r = −0.147, *p* < 0.05) as well as between testosterone and task cohesion (r = −0.230, *p* < 0.01) levels. Additionally, a multiple forward stepwise regression analysis highlighted that cortisone variation might be partially explained by task cohesion; the DHEA—determined by psychological (un)safety in the group, attitude towards the military and military service, and norm cohesion; and the testosterone—determined by task cohesion and adaptation to the new military environment. The results of this study suggest that subjective measures of social factors can be used to predict hair steroid hormone levels as objective measures of the chronic stress perceived by conscripts during their basic military training.

## 1. Introduction

There is growing evidence to show that chronic stress, which is caused by social, environmental, and occupational stressors, can severely affect individual wellbeing. In a military context, stressors can come in various forms, including physical and psychological stressors [1]. During compulsory military service, especially in the early stages, conscripts cope with multiple military training social environmental factors [2] such as adjusting to a new military environment, group cohesion, and psychological unsafety, all of which can affect the psychological and physical response in cases that involve unsuccessful or prolonged adaptation, serving as sources of occupational stress. Conducting research into such an environment may provide new insights in terms of better understanding how human hormones respond to external stressors. To avoid stress-induced negative consequences, stress management programmes may be needed. However, a primary requirement for developing effective interventions is identifying reliable biological indicators in an individual’s stress levels.

The hypothalamic–pituitary–adrenal (HPA) axis represents the central stress response system. Stressors come in many types, including social and physical stressors, but all types activate the HPA axis via different pathways [3]. The stress response involves bidirectional communication between the brain and other body systems via neural and endocrine mechanisms [4] and, consequently, can lead to changes in the level of many hormones [5], which may be used in the evaluation of stress levels. The major limitation of blood, salivary, or urine hormone measurements is the rapid daily concentration fluctuations [6,7]. Although the mechanism that involves hormone incorporation into the hair is not fully understood, the measurement of hormone levels in scalp hair is considered a promising non-invasive technique in chronic stress evaluation [7,8,9]. The analysis of steroid hormones in hair is increasingly being used in stress-related research in order to obtain retrospective data on hormone secretion [10,11]. Cortisol, an end-product of the HPA axis, is considered the primary hormone in a stress response mechanism [12,13,14]. However, HPA axis responses can also be affected by steroid hormones such as dehydroepiandrosterone (DHEA) or testosterone [15,16]. DHEA, a precursor of the sex hormone testosterone, and cortisol are the most abundant human adrenal hormones. Together, they tightly coordinate short-term and long-term endocrine stress responses, while also empowering the physiological and behavioural adjustments that are necessary for maintaining homeostasis [17]. Cortisol and DHEA with testosterone have an inverse relationship: while cortisol stimulates, testosterone and its precursor, DHEA, tend to limit the stress response [18,19]. DHEA also facilitates the metabolism of cortisol to the inactive metabolite cortisone [20]. The cortisone concentration in hair has been less well studied; however, it can provide comprehensive information on the cumulative amount of glucocorticoids in the body [21].

The majority of chronic stress studies, including military-related research, have been directed towards the evaluation of hair cortisol levels [13,14,22,23]. The results of many studying focusing on military veterans or active-duty soldiers deployed to war zones support the usefulness of hair cortisol as a biobehavioral marker of chronic stress: hair cortisol levels highly correlate with posttraumatic stress disorder (PTSD) symptom scores [24], and it was found to be predictive of a more significant increase in PTSD symptomatology in soldiers who had experienced new-onset traumatic events [25]. However, the hair cortisol concentration was reported to be unaffected by basic military training, while military training was perceived as stressful but not as a severe life event. [26]. Moreover, no clear relationship exists between perceived stress and hair cortisol [26,27]. Meanwhile, social environmental factors that are present during the military training that conscripts undertake are assumed to have an effect on perceived stress levels. Previous studies indicate adaptability as an important factor to the stress levels of conscripts [28]. It also has been shown that group cohesion can help to reduce negative stress reactions [29]. However, there have been not studies that have focused on a relationship between social environmental factors during the military training that conscripts undergo and biomedical outcome measures such as stress hormones and the data on hair steroid hormones other than cortisol. The analysis of multiple hair steroid hormone concentrations may provide more precise information on long-term stress exposure, while analysis on an association between hair steroid hormones and the subjective factors of social environment manifested at the beginning of compulsory military service may provide information about the missing linkage between perceived stressors and body hormonal reaction.

The above-mentioned findings lead to the hypothesis that hair steroid hormones are associated with military training social environmental factors and can be used as markers to identify the chronic stress levels of conscripts. The aim of this study was to evaluate concentrations of the major hair steroid hormones: cortisol, cortisone, testosterone, and DHEA, and to identify trends in the association between hair steroid hormone levels and the social environmental factors that are encountered during military training, such as adaptation, cohesion, and psychological (un)safety in the group, and the attitude towards military service amongst military conscripts. Our findings consider a portfolio of stress-related factors and contribute towards the expanding knowledge of the factors that affect hair steroid hormone levels.

## 2. Materials and Methods

### 2.1. Study Participants and Data Collection

The open-access program OpenEpi, (version 3.01) was used to calculate the study sample size. After selecting the research significance level alpha = 0.05 and the research power −80%, the preliminary size of the research sample was estimated. The chronic stress prevalence parameter was assumed to be 50%, as the exact prevalence of this indicator in the study population is unknown. As such, this cross-sectional study included a random sample of 185 male conscripts who were aged between eighteen and twenty-six years. During the recruitment process, the conscripts were evaluated to be mentally and physically healthy and, therefore, the study participants represent a sample of healthy young men. Exclusion criteria included the use of synthetic steroids during over the previous three months. The basic characteristics of the study group are presented in Table 1.

The study was approved by the Vilnius Regional Biomedical Research Ethics Committee (protocol No. 2020/10-1275-754). Informed written consent was obtained from all of the participants who were in volved in the study. There was no reimbursement provided for participations. Participants were informed about the possibility of withdrawing from the study at any point.

The information was collected from two battalions of the Lithuanian Armed Forces in November 2020 and August 2021 using two pools of the conscripts from the autumn and spring calls for compulsory military service to avoid the effect of seasonality in the study. The data were collected during the COVID-19 pandemic, specifically in November 2020 and in August 2021, when additional restrictions were being enforced in line with health safety guidelines. The assessments were conducted after the first month of military service. The first months of service are designed for the conscripts to gain basic military skills (practicing with military equipment on the fixed installation and the field exercises); they compose the first part of the nine-month-duration conscription service in Lithuania. During this period, conscripts adapt to the military environment, i.e., to a military order and military team (squad), as well as increase their physical capacity through individual physical training.

Each enrolled individual was asked to complete a set of questionnaires in order for their anthropometric and lifestyle characteristics to be analysed (including their age, education, and smoking status) along with military training social environmental factors. Hair samples were obtained by trained personnel so that tests could be carried out in terms of a steroid hormone concentration measurement and for the collection of anthropometric data (such as height and weight as well as waist and hip circumferences). Hair samples were collected from the posterior vertex region of the head, as close to the scalp as possible, and these samples were stored in foil at room temperature in a dark environment until the analysis could be carried out. Assuming an average hair growth rate of one centimeter per month, a collected one centimeter hair segment would contain steroid hormones deposited over the previous month. Hair steroid hormones analysis was conducted at the laboratories of the Institute of Biomedical Sciences, Faculty of Medicine, Vilnius University.

### 2.2. Hair Steroid Hormones Analysis

Hair steroid hormones (cortisol, cortisone, DHEA, and testosterone) were determined from the first centimetre of hair proximal to the scalp, representing approximately one month of growth before the hair was sampled. Hair washing and steroid hormone extraction procedures were carried out using the modified method proposed by Gao et al. [10]. A 20 mg hair sample was washed by being gently shaken in 3 mL of isopropanol for three minutes at room temperature. Samples were allowed to dry under the fume hood for at least 24 h. Then, 1.4 mL of methanol and 20 μL of internal standard (cortisol-d4: 50 ng/mL, cortisone-d8: 50 ng/mL, DHEA-d6: 50 ng/mL, testosterone-d3: 10 ng/mL) were added, and the samples were incubated at 50 °C for four hours. The samples were centrifuged at 10,000 rpm for five minutes, and the clear supernatant was transferred into the polypropylene tube. A stream of nitrogen gas was used for the evaporation of methanol and to dry the samples. The dry residue was re-suspended in 200 μL of methanol/water containing 0.05% acetic acid at a ratio of 100:100 (*v*/*v*). The chromatographic separation was carried out on the ultra-high performance liquid chromatography (UHPLC) system coupled with a triple quadrupole tandem mass spectrometer, which was equipped with an electrospray ionisation source and was operated in the positive ionisation mode. Two ion pairs were selected for each analyte, with the most sensitive transition being used for quantification and the rest being used for confirmation. A description of the UHPLC-MS/MS system (Shimadzu Corporation, Kyoto, Japan) and the analysis conditions are presented in Table 2. Data acquisition was conducted using the Shimadzu LabSolutions software (version 1.20).

### 2.3. Military Social Environment-Related Measures

Military social environment-related measures were obtained using a military conscription-specific questionnaire. The items showed good reliability in the study sample: the calculated Cronbach’s alpha coefficients for each item varied from 0.767 to 0.899. The average variance extracted (AVE) and composite reliability (CR) indexes were analyzed to fit the validity of the military environment-related measures. All of the assessed AVE (ranging from 0.526 to 0.627) and CR (ranging from 0.878 to 0.927) were considered. The final version of the questionnaire contained a total of forty-six questions, which served to measure six dimensions:

Attitude towards the military and military service (ATM): This is a six-item inventory that was designed to evaluate attitudes regarding the military in general. Attitude is a strong determinant of commitment as well as being a significant predictor of perceived stress [30]. The items in the inventory were determined based on the research by Salo [31] on Finnish conscript service, which aimed to measure how much military service made sense to the conscripts. Construct values vary in the interval from 6 to 42, where a higher value indicates a more positive attitude towards military service. The calculated Cronbach’s alpha was 0.811, CR was 0.878, and AVE was 0.574.

Adaptation to the new military environment (ADJ): This forms a brief seven-item self-reporting inventory that was used to assess how effective the adaptation can be to the new environment. The items were taken from Salo [31]. They served to measure how conscripts got used to the new military environment and how conscripts adapted to the new military training environment when they were in an environment without their friends and family. Construct values vary in the interval from 7 to 49, where a higher value indicates better adaptation. The calculated Cronbach’s alpha was 0.872; CR was 0.909; and AVE was 0.590.

Team cohesion (CTE): This is a twelve-item inventory that was designed to evaluate the relationships between squad members. It includes communications within a group, whether there is a desire to improve together, and how strong that desire might be. The measure was composed of multiple items that were taken from research by Salo [31] regarding Finnish conscript service, from the group cohesion scale that was revised by Treadwell et al. [32], and from research by Ohlsson et al. [33] on multinational military staff exercise. A sample item follows: “Squad soldiers feel comfortable in expressing disagreements within the group”. Construct values vary in the interval from 12 to 84, where a higher value indicates better team cohesion. The calculated Cronbach’s alpha was 0.899; CR was 0.927; and AVE was 0.563.

Task cohesion (CTS): This is an eight-item inventory that was designed to evaluate the attitude of conscripts towards how effective their squads tend to be in terms of carrying out its assigned tasks, in sharing leadership, and in finding non-traditional ways to achieve the set goals. The items that were included in the research by Ohlsson et al. [33] regarding a multinational military staff exercise were adopted to the environment in which the conscripts found themselves. A sample item follows: “The quality of task performance which has been provided by this squad is improving over time”. Construct values vary in the interval from 8 to 56, where a higher value indicates better task cohesion. The calculated Cronbach’s alpha was 0.767; CR was 0.898; and AVE was 0.526.

Norm cohesion (CIN): This is a six-item inventory that was designed to evaluate the existence of formal and informal rules within the squad and to discern any common tolerance in a group towards otherness, which would help in creating a positive environment. A sample item follows: “It would be a concern if the soldier did not take an active part in the squad’s activities”. Construct values vary in the interval from 6 to 42, where a higher value indicates better norm cohesion. The calculated Cronbach’s alpha was 0.877; CR was 0.910; and AVE was 0.627.

Psychological (un)safety in the group (PSY): This is a seven-item inventory that was designed to evaluate marginalisation within the groups (squads). These negative items were taken from Salo [31] and Ohlsson et al. [33], and a sample item follows: “Other conscripts frequently ignore me”. Construct values vary in the interval from 7 to 49, where a lower value indicates a higher level of psychological safety. The calculated Cronbach alpha was 0.890; CR was 0.918; and AVE was 0.616.

All of the items were measured on a seven-point Likert scale. The research was conducted using printed questionnaires which were provided in the Lithuanian language.

### 2.4. Statistical Analysis

All statistical analyses were performed using the IBM SPSS Statistics 27v software. The Shapiro–Wilk test was used to determine if the data were distributed normally. Quantitative variables are presented as mean ± standard deviation (SD) for normally distributed or median (interquartile range) (IQR) for non-normally distributed variables. For the categorical variables, the absolute and relative frequencies were calculated. The level of statistical significance was set at 0.05 for two-tailed testing.

Since the data collected for steroid hormone concentrations tend to violate the assumption of normality, the forward stepwise model selection method for automatic linear modelling (ALM) was chosen for the inferential statistical analysis [34,35]. The ALM procedure helped to avoid the collinearity issues that existed in all of the final designed models and to side-step the well-known limitations of the traditional regression procedure.

The statistical analysis, which was conducted through ALM building, used the hair steroid hormones (cortisol, cortisone, dehydroepiandrosterone, and testosterone) as the target or predictor variables in the separated models, and six variables were chosen as the predictors that served to assess the military social environment factors. Before the modelling process was undertaken, the auto data preparation side of things was completed, with a confidence level ninety-five percent was achieved. Then, the ALM procedure was carried out by means of the forward stepwise technique for model design [36]. In addition, use was made of Akaike’s information criterion corrected (AICC) for the entry or elimination of possible predictors and to choose the most parsimonious model [36]. The importance of the predictors in the models was assessed in a stepwise fashion using incremental R^2^ [37,38]. Repeated analyses were conducted with the random seed of 54752075. Moreover, the validation of the designed models was conducted using the paired samples *t*-test between the measured and predicted data [39,40,41]. The automatic linear modelling analysis results as they concern the descriptions of the four designed models are summarised below in Section 3.2.

Four models were designed: Model 1 involved a prediction of the cortisol levels; Model 2 involved a prediction of the cortisone levels; Model 3 involved a prediction of the dehydroepiandrosterone levels; and Model 4 covered a prediction of the testosterone levels. The six military training social environment factors were used as predictor variables: attitude towards military service (ATM); adaptation to a new military environment (ADJ); team cohesion (CTE); task cohesion (CTS); norm cohesion (CIN); and psychological (un)safety in the group (PSY).

## 3. Results

Predictive models were constructed to clarify the hair steroid hormone levels (cortisol, cortisone, DHEA, and testosterone). The structure and accuracy testing for the constructed models is described in detail below, in Section 3.2. Additionally, the robustness-testing results for the established models are presented in Section 3.3.

### 3.1. Preliminary Analysis 

The descriptive statistics of the stress-related variables is presented in Table 3.

The following significant positive correlations were identified between the different indicators of military training social environmental factors: task cohesion and team cohesion (r = 0.736, *p* < 0.01); norm cohesion and team cohesion (r = 0.577, *p* < 0.01); team cohesion and attitude towards military service (r = 0.540, *p* < 0.01); and norm cohesion and task cohesion (r = 0.516, *p* < 0.01). Significant negative correlations were identified between hair steroid hormone levels and the following military training social environmental factors: cortisol and adaptation to a new environment (r = −0.176, *p* < 0.05) and cortisol and attitude towards military service (r = −0.147, *p* < 0.05). Moreover, a significant negative correlation was identified between testosterone and task cohesion (r = −0.230, *p* < 0.01). These variables were included in the modelling procedures. The relationships in the research variables are presented in Table 4.

### 3.2. Automatic Linear Modelling Results

All six military training social environmental factors were included in the forward stepwise linear regression analysis achieved by ALM modelling to discover which variables were the best predictors for hair steroid hormone levels. Additionally, the hair steroid hormones were included as predictors in the designed models where they were not chosen as a target variable.

#### 3.2.1. The Effects in the Constructed Models, and Building Steps

**Figure A1:**
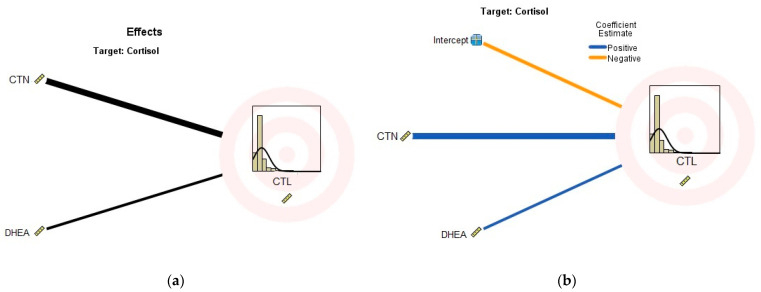
The forward stepwise model selection method results presented for Model 1 after automatic linear modelling: (**a**) the identified effects of the predictors on cortisol levels (CTL) by cortisone (CTN) and dehydroepiandrosterone (DHEA) and (**b**) the negative and positive coefficient estimations that were identified for cortisone (CTN) and dehydroepiandrosterone (DHEA) in Model 1.

**Figure A2:**
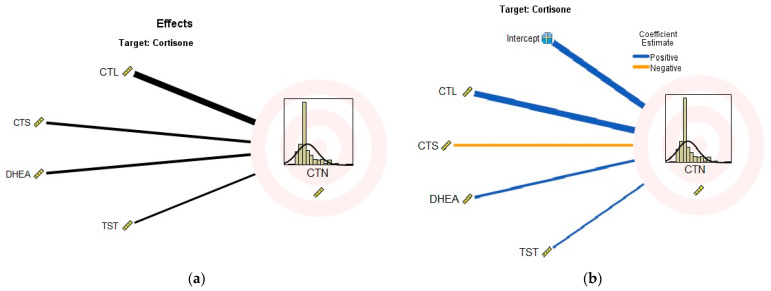
The forward stepwise model selection method results presented for Model 2 after automatic linear modelling: (**a**) the identified effects of the predictors on cortisone (CNT) levels by cortisol (CTL), task cohesion (CTS), dehydroepiandrosterone (DHEA), and testosterone (TST) and (**b**) the negative and positive coefficient estimations that were identified in Model 2 for cortisone (CNT) levels by cortisol (CTL), task cohesion (CTS), dehydroepiandrosterone (DHEA), and testosterone (TST).

**Figure A3:**
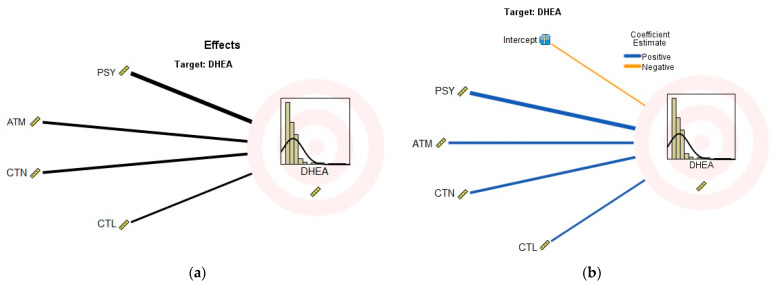
The forward stepwise model selection method results presented for Model 3 after automatic linear modelling: (**a**) the identified effects for the predictors on dehydroepiandrosterone levels (DHEA) by psychological (un)safety in the group (PSY), attitude towards the military service (ATM), cortisone (CTN), and cortisol (CTL) and (**b**) the negative and positive coefficient estimations that were identified in Model 3 for psychological (un)safety in the group (PSY), attitude towards the military service (ATM), cortisone (CTN), and cortisol (CTL).

**Figure A4:**
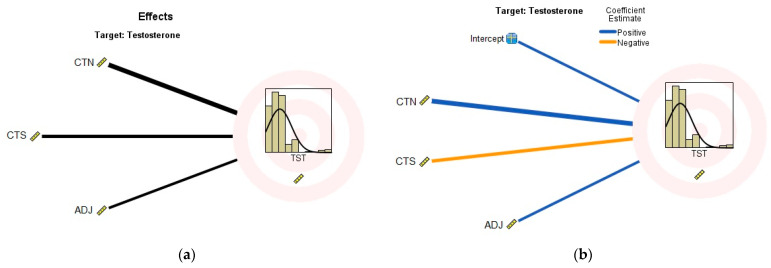
The forward stepwise model selection method results presented for Model 4 after automatic linear modelling: (**a**) the identified effects for the predictors on testosterone (TST) levels by cortisone (CTN), task cohesion (CTS), and adaptation to the new military environment (ADJ) and (**b**) the negative and positive coefficient estimations that were were identified in Model 4 for cortisone (CTN), task cohesion (CTS), and adaptation to the new military environment (ADJ).

Forward stepwise linear regression analysis was used to design the models. In the constructed models of all of the predictors were included, with the *p*-value effects being less than 0.05 and being removed with the effects when the *p*-value was greater than 0.1. For the opportunity to be able to repeat the modelling analyses with the same settings, a random seed was set (a pseudo-random integer number, 54752075). The details of the effects and building steps being used in the constructed models with validation by AICC are presented in Table 5. Additionally, diagrams presenting the effects of the predictors for all of the constructed models can be found in the Back Matter of this paper (see Figure A1a, Figure A2a, Figure A3a and Figure A4a in Appendix A).

The F Statistics indicate that all four models have a high level of accuracy (see Model 1, Table 4). The model for cortisol (Model 1) revealed that cortisone and DHEA were the best predictors for cortisol levels (see Model 1, Table 5, AICC = 466.614). However, despite the preliminary analyses, a significant negative correlation was identified between cortisol levels and military training social environmental factors such as adaptation to a new military environment and attitude towards military service (see Table 4); these variables did not show any significant effects and were rejected from Model 1.

The model for cortisone (Model 2) revealed that four factors—cortisol, task cohesion, DHEA, and testosterone, showed the effects for an overall model (see Model 2, Table 5, AICC = 614.314). Though task cohesion did not correlate with cortisone, it was included in the Model 2 training set in the second modelling step, and an important effect was identified upon the prediction of cortisone as a military training social environmental factor.

Two military training social environmental factors were chosen as the best predictors for the DHEA levels (Model 3), psychological (un)safety in the group (PSY) and attitude towards military service (see Model 3, Table 5, AICC = 868.334). It is important to reference the fact that the predictor psychological (un)safety in the group was included in the Model 3 training set in the first modelling step, and it identified a highly important effect upon DHEA prediction levels.

In the model for testosterone (Model 4), the significant effects were identified for three selected predictors. The significant F Statistics and the information criterion AICC = −248.350 revealed that cortisone and two military training social environmental factors—task cohesion and adaptation to a new military environment, were the best predictors for testosterone levels (see Model 4, Table 5).

#### 3.2.2. Coefficient and Predictor Importance in the Constructed Models

Additionally, calculations were conducted to determine the standardised beta coefficient values, significance t statistic tests, and 95% confidence intervals for the individual model coefficients. All of the models are presented the same way: after the intercept the independent variable effects were organised from top to bottom by decreasing the predictor importance of each of the parameters that are included in the model (see Table 6). Additionally, within the predictors that contain effects, the coefficients are organised by the ascending order of data value. The calculated coefficients for Model 1, Model 2, Model 3, and Model 4 are presented in Table 6. Additionally, diagrams are presented for all of the constructed models (see Figure A1b, Figure A2b, Figure A3b, and Figure A4b in Appendix A).

### 3.3. Robustness Testing for the Established Models

The descriptive statistics for paired data samples in terms of the determined and predicted steroid hormone levels are presented in Table A1, Appendix B. A significant positive correlation was found between predicted cortisol levels (r = 0.95, *p* < 0.01), cortisone levels (r = 0.95, *p* < 0.01), dehydroepiandrosterone levels (r = 0.95, *p* < 0.01), and testosterone levels (r = 0.95, *p* < 0.01) (see Table A2, Appendix B). The details for the paired samples *t*-test are presented in Table 7.

The conducted *t*-test statistics for the paired samples verified the fact that there is no average difference between the measured cortisol levels and the predicted levels in Model 1 (*t*_183_ = 0.785, *p* = 0.434), between the measured cortisone levels and the Model 2 predicted levels (*t*_182_ = −0.182, *p* = 0.856), between the measured dehydroepiandrosterone levels and the levels predicted by Model 3 (*t*_182_ = 0.235, *p* = 0.814), or the measured testosterone levels and the figures that were predicted by Model 4 (*t*_182_ = 1.274, *p* = 0.204).

According to the average of the determined and foreseen hair steroid hormone levels, it can be seen that the detected steroid levels are equivalent to the predicted levels as follows:

1. The measured cortisol levels were similar to the predicted levels in Model 1, with 95% of a confidence interval CI ∈ (−0.196, 0.454);
2. The measured cortisone levels were similar to the predicted levels in Model 2, with 95% of a confidence interval CI ∈ (−0.453, 0.377);
3. The measured dehydroepiandrosterone levels were similar to the predicted levels in Model 3, with 95% of a confidence interval CI ∈ (−1.009, 1.282);
4. The measured testosterone levels were similar to the predicted levels in Model 4, with 95% of a confidence interval CI ∈ (−0.07, 0.077).

The outcomes from the applied *t*-test statistics for the paired samples proved the robustness of the designed models, with an insignificant difference between the measured and predicted data being observed.

## 4. Discussion

This study aimed to analyse the hormonal profile of the hair of conscripts in the early stages of their service while investigating the potential relationships with military training social environmental factors in order to identify the reliable biological indicators of an individual’s stress level. The association between these objective and subjective measures is important to understand how the military social environment could be improved at the beginning of basic military training to avoid significant effects on hormone activation as a side effect of conscription service.

Our study used scalp hair analysis to evaluate steroid hormone levels rather than the more conventional methods such as serum, saliva, or urine measurements [7,9]. This method makes it possible to determine long-term steroid levels [10,11] and eliminates any effect of daily hormone level fluctuations, especially in terms of the influence of stress, especially stress that has been caused by invasive sampling procedures [6,7]. In addition, it is essential to indicate the fact that scalp hair samples are easy to collect and are relatively stable [40].

Amongst other steroid hormones, hair cortisol is widely used as the primary biomarker of long-term exposure to chronic stress in a broad spectrum of psychoneuroendocrinological studies [9,13,14]. Increased hair cortisol levels were found in various contexts (e.g., endurance athletes [42,43,44], shift work [21], sleep and mental disorders [45], unemployment [46], chronic pain [47], or major life events [48]). Long-term elevations in serum [49] and hair cortisol [50] have been also reported during stressful military captivity training. Meanwhile, longitudinal research on basic military training at the beginning of military service reports controversial findings: in Boesh et al. [26], research on Swiss conscripts reports that basic military training has no effect on cortisol concentration, while Gifford et al. [50] conducted research on UK cadets and indicated an increase in the cortisol concentration during basic military training. Within the findings of our study, we can confirm an association between cortisol and external stress factors during military conscription: weak but significant negative correlations were observed between cortisol and individual adjustment to a new military environment (r = −0.176, *p* < 0.05) as well as between cortisol and individual attitude towards military service (r = −0.147, *p* < 0.05), which indicates that long-term cortisol hypersecretion in youth emerges more from individual factors, such as adjustment and attitude, than it does from group cohesion factors (team, task, and norm cohesion).

An evaluation of a broader spectrum of hair steroid hormones levels, especially when sampled under low-stress conditions, should be something that is thought of as being debatable, as stress affects not only the activity of the hypothalamic–pituitary–adrenal axis but also the activity of the hypothalamic–pituitary–gonadal axis [15,16]. Despite this demand, to our knowledge, no studies of multiple hair steroid hormone concentrations in terms of correlations with subjective measures of social factors have yet been performed on military cohorts. Our research results indicate that other steroid hormones, such as hair cortisone, DHEA, and testosterone, which are not typically related to social environment and that have been identified as a primary biomarker of long-term exposure to chronic stress, can be explained by military training social environmental factors. Weak but significant negative correlations between testosterone levels and task cohesion (r = −0.230, *p* < 0.01) indicate that variation in the testosterone level is linked to the group cohesion factor. Furthermore, by combining hormones and social environmental factors into one statistical model and applying an automatic linear modelling algorithm, it is confirmed that cortisone, DHEA, and testosterone levels can be explained by variation in the perceived social environmental factors during conscription to some extent. Task cohesion was identified as a marker for the prediction of lower hair cortisone levels (Model 2, CTS importance = 0.048) and testosterone levels (Model 4, CTS importance = 0.307). Adjustment to a new military environment can also be a predictor for testosterone levels (Model 4, ADJ importance = 0.191). Psychological (un)safety in the group (PSY) as well as attitude towards military service (ATM) were both recognised as significant markers for DHEA concentration levels (Model 3, PSY importance = 0.458 and ATM importance = 0.207).

In order to explain these findings, the possibility of a bidirectional relationship between social factors and steroid hormones has to be taken into account. Subjectively perceived social factors influence individual hormonal response, and the steroid hormones modulate behavior through the corticosteroid receptors that are present in the brain. During the first month of basic military training, the conscript transitions from their home environment to a process of adjusting to military rules and regulations, acquiring a new skill set, building up a new network, and becoming part of a unit along the way [51]. Therefore, any interplay between individual military organisational factors and the biopsychosocial vulnerability of individuals may affect an individual’s stress response, where it is coordinated through hormone action.

Similarly, the presence of increased DHEA in response to an acute psychosocial stressor [52] is something that has been found by other researchers. Acute stress lowers testosterone concentration, but on the whole, this is temporary [53,54,55,56] and can help one to adapt to stressful situations; on the other hand, lower levels of testosterone under conditions that involve chronic stress often correlate with a higher risk of PTSD [57,58]. However, with the exception of cortisol levels, the aforementioned findings come from studies that involve blood and saliva hormone measurements. There is a lack of research using human subjects that includes hair steroid hormone profiles when evaluating long-term stress exposure due to psychosocial factors. It is worth mentioning a result from the previous study that was conducted using laboratory mice, in which a higher level of DHEA—but not testosterone—was found in mice who had been housed in groups against those which had been housed in pairs [59]. This finding is in line with the fact that DHEA is a neuroactive steroid with action responses at several neurotransmitter receptors [60], indicating the importance of DHEA level evaluation in the psychosocial context.

The study was conducted during the peak of the COVID-19 pandemic, when military service was being organised in isolation from external contact. From that point of view, it is essential to point out the fact that some cultural or social support factors that would normally have allowed a conscript to be connected to friends or family were excluded during this period, so other military factors such as task cohesion or high levels of motivation to undertake one’s service period became protective factors for stress accumulation. The main study strengths of the present study include the possibility of being able to eliminate the effect of non-military service-related stressors to a maximum along with validated scales with high levels of reliability and the automatic linear modelling procedure. An additional advantage in the use of this study was the fact that we were able to analyse hair steroid hormone levels using UHPLC-MS/MS. Liquid chromatography and tandem mass spectrometry is superior to immunoassays (IAs) for this purpose since despite sensitivity, cost, and simplicity, IAs include cross reactivity with similar analytes along with the existing standardisation issues between labs [61].

This study had several limitations. First, the absence of any female participants could be indicated as the main weakness of our study. Our results should be confirmed in more heterogeneous populations in order to generalise our findings across women or other age groups. Second, it is also essential to indicate that due to the abundance of modifications and a lack of standardised methodology [7] for the analysis of hair steroid hormone concentrations, any interpretation of the results remains complicated. The same can be said about military training social environmental factors measurements. Due to the specifics of the conscription service and the limited accessibility to the conscript population, there are not many studies investigating conscription-specific social environmental factors and their effects. Furthermore, there are no standardized and cross-study validated tests to measure stress-related social environment factors in the military, such as adaptation to the military environment or elements of the conscripts’ squad cohesion. In this study, we used questionnaires developed by previous researchers in their military and conscription studies, but this limits the compatibility of this research results with the research results obtained in other (non-military and non-military conscription) settings. Third, the cohort of military conscripts is specific because of the conclusory nature of the service; therefore, the interpretation of the results for another military cohort may be less comparable. Furthermore, due to the cross-sectional study design, we cannot determine the causality in the relationships between the hair steroid hormone levels and the military training social environmental factors.

## 5. Conclusions

The study revealed the significant effects of military training social environmental factors on hair cortisone, DHEA, and testosterone levels. Task cohesion, adaptation to a new military environment, psychological (un)safety in the group as well as attitude towards military and military service were shown to have a significant effect on hair steroid hormone levels in conscripts. These study findings suggest that cortisol could be considered as an objective biomarker for chronic stress that may be caused by compulsory conscript service and that cortisone, DHEA, and testosterone can also be included alongside it. Our study continues in terms of creating more precise modelling for chronic stress predictors, including the task of analyzing the ratios between individual hair steroid hormones in a longitudinal study.

## Figures and Tables

**Table 1:** Anthropometric values and lifestyle details of the study participants.

Characteristic	Value
Age (years), median (IQR)	20.32 (1.61)
Education, n (%)	
Unfinished secondary	10 (5.3)
Secondary	134 (71.4)
Vocational school	29 (17.0)
Higher education (university or non-university)	12 (6.4)
Height (m), mean (SD)	183.19 (7.07)
Weight (kg), mean (SD)	79.79 (11.79)
Body mass index (kg/m^2^), median (IQR)	24.00 (16)
Waist-to-hip ratio, median (IQR)	0.84 (1.04)
Smoking status, n (%)	
Yes	78 (42.0)
Occasionally	48 (26.0)
No	59 (32.0)
Hair dyeing over last three months, n (%)	
Yes	3 (1.6)
No	182 (98.4)
Hair washing frequency, n (%)	
Once a week or less	3 (1.2)
Between 2–4 times a week	61 (33.1)
Five times a week and more	121 (65.7)

Notes: descriptive statistics of normally distributed quantitative measures have been described using mean and standard deviation (SD), while non-normally distributed variables were presented as median and interquartile range (IQR).

**Table 2:** Ultra-high performance liquid chromatography-tandem mass spectrometry (UHPLC-MS/MS) system and analysis conditions.

UHPLC-MS/MS System Components (Shimadzu Corporation, Kyoto, Japan)	
Solvent delivery units (binary pumps) LC-30AD Autosampler SIL-30AC Column oven CTO-20AC Triple quadrupole tandem mass spectrometer LCMS-8060	
UHPLC column YMC-Triart Bio C4 (3.0 × 100 mm, 1.9 µm)	
Chromatographic separation conditions	
Column temperature	50 °C
Mobile phase	methanol and water acidified with 0.05% acetic acid (binary gradient)
Flow rate	0.4 mL/min
Injection volume	10 µL

**Table 3:** The descriptive analysis results for the gathered dataset.

Variables ^1^	Mean		Dispersion			Distribution			
	Statistic	Std Error	Std Deviation	Min	Max	Skewness		Kurtosis	
						Statistic	Std Error	Statistic	Std Error
CTL	5.224	0.331	4.483	1.034	31.597	3.041	0.179	11.126	0.356
CTN	16.847	0.554	7.518	3.233	48.204	1.818	0.179	4.008	0.356
DHEA	13.860	0.851	11.541	2.912	77.368	3.203	0.179	12.182	0.356
TST	0.645	0.039	0.528	0.143	4.133	3.324	0.179	14.672	0.356
ADJ	35.380	0.667	9.047	10	49	−0.719	0.179	0.112	0.356
ATM	28.761	0.604	8.197	6	42	−0.810	0.179	0.258	0.356
CTE	61.935	0.958	12.995	22	84	−0.643	0.179	0.227	0.356
CTS	39.234	0.487	6.600	22	52	−0.541	0.179	−0.211	0.356
CIN	31.870	0.567	7.697	8	42	−0.987	0.179	0.509	0.356
PSY	15.891	0.603	8.174	7	44	1.179	0.179	1.112	0.356

^1^ Valid N (listwise) = 184. Abbreviations used: cortisol (CTL), cortisone (CTN), dehydroepiandrosterone (DHEA), testosterone (TST), attitude towards the military service (ATM), adaptation to the new military environment (ADJ), team cohesion (CTE), task cohesion (CTS), norm cohesion (CIN), and psychological (un)safety in the group (PSY).

**Table 4:** The relationship between hair steroid hormone levels and military training social environmental factors.

	CTL	CTN	DHEA	TST	ADJ	ATM	CTE	CTS	CIN	PSY
**CTL**	1.000	0.726 **	0.388 **	0.306 **	−0.176 *	−0.147 *	−0.108	−0.117	−0.123	0.103
**CTN**	0.726 **	1.000	0.385 **	0.339 **	−0.127	−0.109	−0.070	−0.135	−0.044	0.056
**DHEA**	0.388 **	0.385 **	1.000	0.382 **	−0.057	0.016	−0.041	−0.035	−0.074	0.121
**TST**	0.306 **	0.339 **	0.382 **	1.000	0.029	−0.089	−0.109	−0.230 **	−0.110	0.127
**ADJ**	−0.176 *	−0.127	−0.057	0.029	1.000	0.480 **	0.361 **	0.396 **	0.445 **	−0.433 **
**ATM**	−0.147 *	−0.109	0.016	−0.089	0.480 **	1.000	0.540 **	0.484 **	0.358 **	−0.502 **
**CTE**	−0.108	−0.070	−0.041	−0.109	0.361 **	0.540 **	1.000	0.736 **	0.577 **	−0.604 **
**CTS**	−0.117	−0.135	−0.035	−0.230 **	0.396 **	0.484 **	0.736 **	1.000	0.516 **	−0.557 **
**CIN**	−0.123	−0.044	−0.074	−0.110	0.445 **	0.358 **	0.577 **	0.516 **	1.000	−0.608 **
**PSY**	0.103	0.056	0.121	0.127	−0.433 **	−0.502 **	−0.604 **	−0.557 **	−0.608 **	1.000

Note: Spearman’s “r” correlation is significant at the * *p* < 0.05 or ** *p* < 0.01 level (a two-tailed test). Abbreviations used: cortisol (CTL), cortisone (CTN), dehydroepiandrosterone (DHEA), and testosterone (TST) attitude towards the military service (ATM), adaptation to the new military environment (ADJ), team cohesion (CTE), task cohesion (CTS), norm cohesion (CIN), psychological (un)safety in the group (PSY).

**Table 5:** Forward stepwise model effects and validation by AICC description.

Source	Sum of Squares	df	Mean Square	F	*p*	Model Building Steps and Validation by AICC			
						1	2	3	4
**Model 1: Target = Cortisol**									
Corrected Model	1430.698	2	715.349	57.611	0.000	469.236	466.614	----	----
CTN	951.591	1	951.591	76.637	0.000	√	√	----	----
DHEA	58.011	1	58.011	4.672	0.032		√	----	----
Residuals	2247.439	181	12.417						
Corrected total	3678.138	183							
**Model 2: Target = Cortisone**									
Corrected model	5324.098	4	1331.025	47.731	0.000	627.452	620.991	617.747	614.314
CTL	2877.989	1	2877.989	103.207	0.000	√	√	√	√
CTS	158.801	1	158.801	5.695	0.008	----	√	√	√
DHEA	152.771	1	152.771	5.478	0.020	----	----	√	√
TST	95.613	1	95.613	3.429	0.066	----	----	----	√
Residuals	4963.654	178	27.886						
Corrected total	10,287.752	182							
**Model 3: Target = DHEA**									
Corrected model	4395.288	4	1098.822	9.833	0.000	877.001	872.618	869.959	868.334
PSY	1189.803	1	1189.803	10.648	0.001	√	√	√	√
ATM	537.447	1	537.447	4.810	0.030	----	√	√	√
CTN	459.050	1	59.050	4.108	0.044	----	----	√	√
CTL	410.647	1	410.647	3.675	0.057	----	----	----	√
Residuals	19,890.228	178	111.743						
Corrected total	24,285.516	182							
**Model 4: target = testosterone**									
Corrected model	5.780	3	1.927	7.659	0.000	−244.354	−246.036	−248.350	----
CTN	2.879	1	2.879	11.441	0.001	√	√	√	----
CTS	1.757	1	1.757	6.985	0.009	----	√	√	----
ADJ	1.097	1	1.097	4.360	0.038	----	----	√	----
Residuals	45.035	179	0.252						
Corrected total	50.815	182							

Notes: Model 1 = the response variable is cortisol; Model 2 = the response variable is cortisone; Model 3 = the response variable is dehydroepiandrosterone; Model 4 = the response variable is testosterone; F = F-test statistic; *p* = significance value; √ = the variable which was included into the model at a specific step; AICC = the effect of the variable that was included into the model at a specific step. Abbreviations used: cortisol (CTL), cortisone (CTN), dehydroepiandrosterone (DHEA), and testosterone (TST) attitude towards the military service (ATM), adaptation to the new military environment (ADJ), team cohesion (CTE), task cohesion (CTS), norm cohesion (CIN), psychological (un)safety in the group (PSY).

**Table 6:** Calculated coefficients description for the designed models.

Model Name ^1^	Coefficient β	Std Error β	t	*p*	Confidence Interval 95%		Importance
					Lower	Upper	
**Model 1: Target = Cortisol**							
Intercept	−2.068	0.737	−2.805	0.006	−3.523	−0.613	
CTN	0.369	0.042	8.754	0.000	0.286	0.452	0.943
DHEA	0.093	0.043	2.161	0.032	0.008	0.178	0.057
**Model 2: Target = Cortisone**							
Intercept	12.285	2.828	4.344	0.000	6.704	17.866	
CTL	1.455	0.143	10.159	0.000	1.173	1.738	0.806
CTS	−0.347	0.062	−2.863	0.008	−0.269	−0.005	0.048
DHEA	0.154	0.066	2.341	0.020	0.024	0.283	0.047
TST	2.350	1.269	1.852	0.066	−0.154	4.855	0.029
**Model 3: Target = DHEA**							
Intercept	−7.203	4.608	−1.563	0.120	−16.296	1.890	
PSY	1.368	0.113	9.263	0.001	0.145	0.590	0.458
ATM	0.233	0.106	2.193	0.030	0.023	0.443	0.207
CTN	0.321	0.159	2.027	0.044	0.008	0.634	0.177
CTL	0.672	0.350	1.917	0.057	−0.020	1.363	0.158
**Model 4: Target = Testosterone**							
Intercept	0.643	0.276	2.328	0.021	0.098	1.188	
CTN	0.190	0.006	3.382	0.001	0.108	0.230	0.502
CTS	−0.107	0.006	−2.643	0.009	−0.129	−0.004	0.307
ADJ	0.100	0.005	2.088	0.038	0.091	0.209	0.191

Notes: ^1^ Model 1 = the response variable is cortisol; Model 2 = the response variable is cortisone; Model 3 = the response variable is dehydroepiandrosterone; Model 4 = the response variable is testosterone; β = standardised beta coefficient; “Std error” β = the standard error of the beta coefficient; t = *t*-test statistic; *p* = significance value; “Importance” = the importance of the effect the variable is associated with the response/target variable. Abbreviations used: cortisol (CTL), cortisone (CTN), dehydroepiandrosterone (DHEA), and testosterone (TST) attitude towards the military service (ATM), adaptation to the new military environment (ADJ), team cohesion (CTE), task cohesion (CTS), norm cohesion (CIN), and psychological (un)safety in the group (PSY).

**Table 7:** Details of the conducted paired samples *t*-test so that differences can be assessed.

Pair	Paired Differences							
	Mean	SD	Std Error Mean	CI 95%		*t*-test		
				Lower	Upper	t	df	*p*
Pair 1	0.129	2.233	0.165	−0.196	0.454	0.785	183	0.434
Pair 2	−0.038	2.852	0.210	−0.453	0.377	−0.182	182	0.856
Pair 3	0.137	7.854	0.581	−1.009	1.282	0.235	182	0.814
Pair 4	0.030	0.323	0.024	−0.017	0.077	1.274	182	0.204

Notes: Pair 1 measured cortisol levels and predicted levels in Model 1; Pair 2 measured cortisone levels and predicted levels in Model 2; Pair 3 measured dehydroepiandrosterone levels and predicted levels in Model 3; and Pair 4 measured testosterone levels and predicted levels in Model 4. The 95% CI referenced a 95% confidence interval for the difference; the *t*-test relates to the student *t*-test; df denotes degrees of freedom; and *p* represents for the statistical significance which is two-tailed tested.

## Data Availability

The data supporting the reported results have been archived in the National Open Access Research Data Archive (MIDAS) at www.midas.lt, accessed on 10 November 2021.

## Appendix B

**Table A1:** Paired samples statistics for measured and predicted levels of stress-related hormones.

Pairs Descriptions		N	Mean	Std Error Mean	Std Deviation
Pair 1	CTL	183	5.224	0.331	4.483
	Model 1 (CTL)	183	5.354	0.244	3.316
Pair 2	CTN	182	16.804	0.554	7.519
	Model 2 (CTN)	182	16.765	0.466	6.316
Pair 3	DHEA	182	13.911	0.854	11.551
	Model 3 (DHEA)	182	14.048	0.592	8.011
Pair 4	TST	182	0.645	0.039	0.528
	Model 4 (TST)	182	0.675	0.029	0.390

Notes: Pair 1 measured cortisol levels and predicted levels in Model 1 (CTL); Pair 2 measured cortisone levels and predicted levels in Model 2 (CTN); Pair 3 measured dehydroepiandrosterone levels and predicted levels in Model 3 (DHEA); Pair 4 measured testosterone levels and predicted levels in Model 4 (TST) levels.

**Table A2:** Correlations of paired sample stress-related hormones.

Pairs Description		N	Correlation Coefficient	*p*
Pair 1	CTL	184	0.878	0.000
	Model 1 (CTL)			
Pair 2	CTN	183	0.930	0.000
	Model 2 (CTN)			
Pair 3	DHEA	183	0.734	0.000
	Model 3 (DHEA)			
Pair 4	TST	183	0.793	0.000
	Model 4 (TST)			

Notes: Pair 1 measured cortisol levels and predicted levels in Model 1 (CTL); Pair 2 measured cortisone levels and predicted levels in Model 2 (CTN); Pair 3 measured dehydroepiandrosterone levels and predicted levels in Model 3 (DHEA); Pair 4 measured testosterone levels and predicted levels in Model 4 (TST). *p* = statistical significance two-tailed tested.
